# Construction of a Prognostic Risk Prediction Model for Obesity Combined With Breast Cancer

**DOI:** 10.3389/fendo.2021.712513

**Published:** 2021-09-09

**Authors:** Na Sun, Dandan Ma, Pingping Gao, Yanling Li, Zexuan Yan, Zaihui Peng, Fei Han, Yi Zhang, Xiaowei Qi

**Affiliations:** ^1^Department of Breast and Thyroid Surgery, Southwest Hospital, Army Medical University, Chongqing, China; ^2^Institute of Pathology and Southwest Cancer Center, Key Laboratory of the Ministry of Education, Southwest Hospital, Army Medical University, Chongqing, China; ^3^Institute of Toxicology, College of Preventive Medicine, Army Medical University, Chongqing, China

**Keywords:** obesity, breast cancer, TCGA, GEO, prognostic model

## Abstract

The improvement in the quality of life is accompanied by an accelerated pace of living and increased work-related pressures. Recent decades has seen an increase in the proportion of obese patients, as well as an increase in the prevalence of breast cancer. More and more evidences prove that obesity may be one of a prognostic impact factor in patients with breast cancer. Obesity presents unique diagnostic and therapeutic challenges in the population of breast cancer patients. Therefore, it is essential to have a better understanding of the relationship between obesity and breast cancer. This study aims to construct a prognostic risk prediction model combining obesity and breast cancer. In this study, we obtained a breast cancer sample dataset from the GEO database containing obesity data [determined by the body mass index (BMI)]. A total of 1174 genes that were differentially expressed between breast cancer samples of patients with and without obesity were screened by the rank-sum test. After weighted gene co-expression network analysis (WGCNA), 791 related genes were further screened. Relying on single-factor COX regression analysis to screen the candidate genes to 30, these 30 genes and another set of TCGA data were intersected to obtain 24 common genes. Finally, lasso regression analysis was performed on 24 genes, and a breast cancer prognostic risk prediction model containing 6 related genes was obtained. The model was also found to be related to the infiltration of immune cells. This study provides a new and accurate prognostic model for predicting the survival of breast cancer patients with obesity.

## Introduction

According to the World Health Organization (WHO), a person with a body mass index (BMI) equal to or greater than 30 kg/m2 is considered obese (1). According to this definition, some epidemiological studies have shown a significant increase in the number of obese individuals in the last decades (2, 3). Obesity is considered as an indicator of metabolic syndrome (MetS). The presence of MetS can increase the risk and influence the prognosis of various tumors, such as breast cancer, colorectal cancer, and prostate cancer (4–6). Recent studies have demonstrated that overweight and obesity are associated with higher risks of adenocarcinoma of the esophagus, thyroid, pancreas, colon, rectum, endometrium, prostate, gallbladder, ovary, and breast, in addition to multiple myeloma (7). Breast cancer is the second leading cause of cancer related deaths in women; therefore, it is of great public health significance to understand how obesity affects this disease. Several studies have identified obesity as a risk factor for breast cancer and are associated with different types of breast cancer, also depending on different stages or ages (8, 9). In addition, obesity has been identified as a poor prognostic factor for breast cancer. For example, several studies have established obesity as a risk factor for postmenopausal breast cancer, specifically estrogen receptor-positive and triple-negative phenotypes.

With the emergence and rapid development of chip and sequencing technologies in various tumors, bioinformatics analysis has been widely used to identify more effective potential biomarkers for the diagnosis, treatment, and prognosis of a variety of diseases. Over the past decade, the Gene Expression Omnibus (GEO) and Cancer Genome Atlas (TCGA) databases have accumulated abundant genomes and gene expression profiles that can be used in various diseases. Weighted gene co-expression network analysis (WGCNA) is a systems biology method used to describe the correlation patterns between genes in microarray samples. WGCNA can be used to find highly correlated gene modules, and the identified correlation networks facilitate network-based gene screening methods that can be used to identify candidate biomarkers or therapeutic targets. WGCNA has been successfully used to study cancer-related targeting modules and central genes (10, 11). Polygenic combinations have been reported to possess better predictive ability than single genes for cancer prognosis (12). Therefore, novel biological algorithms need to be explored to construct more accurate diagnostic or prognostic models.

In this study, we used public microarray expression to comprehensively analyze breast cancer patient data from the Gene Expression Omnibus (GEO) and The Cancer Genome Atlas (TCGA) databases, and modules associated with obesity were identified by WGCNA. Cox and LASSO regression models were used to construct a risk score prediction model, which could help better predict the prognosis of breast cancer patients with obesity.

## Materials and Methods

### Data Resource

We downloaded the expression profile data and sample information of GSE24185 using the GEOquery package (13) in R software version 4.0 (http://www.r-project.org), and downloaded the corresponding GPL96 chip information of expression profile data. The chip information in the expression profile data was converted into gene symbol, and part of the data without the gene symbol information was removed during the conversion, and the duplicate data were averaged. We used the cgdsr package in R software to download the required breast cancer gene expression data and sample clinical information. In the GSE24185 dataset, we selected data from 74 breast cancer patients, including 38 obese samples (BMI >30 Kg/m^2^) and 36 normal samples (BMI 18.5-24.9 Kg/m^2^). Another TCGA dataset was downloaded from Xena using the UCSCXenaTools package for validation.

### Enrichment Analysis and Weighted Gene Co-Expression Network Analysis

Principal component analyses (PCA) were performed using the R software. GO and KEGG enrichment analyses were performed using clusterProfiler package (14) in R software version 4.0, and enrichment pathways with statistical significance were screened under the conditions of *p* < 0.05 and *q* < 0.2. The metabolic syndrome is a common metabolic disorder. Obesity is considered as an indicator of MetS. Co-expression analysis of the resulting differentially expressed genes was performed using WGCNA package (15) in R. Finally, modules with higher correlation with obesity were identified based on correlation coefficient and the modules with statistical significance were screened under the conditions of *p* < 0.05.

### COX Proportional Hazard Model

Compared with the traditional stepwise regression model, the lasso regression model has the advantage of processing all the independent variables at the same time, which greatly strengthens the stability of the model, yielding a model with fewer variables at a faster speed. Lasso regression analysis was performed by glmnet package (16) in R.

### Immune Score and Matrix Score

CIBERSORT is a deconvolution algorithm that uses a feature matrix of 547 genes to represent 22 infiltrating immune cells (17, 18). CIBERSORT uses Monte Carlo sampling to derive a deconvoluted p value for each sample, and deletes samples with *P* > 0.05. The ESTIMATE algorithm (estimate package in R 4.0 software) was used to calculate the immune and matrix scores of each tumor sample, and the correlation between tumor and obesity was analyzed according to the matrix score.

## Results

### Stromal and Immune Scores

The expression profile data and sample information of GSE24185 were downloaded using the GEOquery package, and the GPL96 chip information corresponding to the expression profile data was downloaded. The samples were subjected to stromal and immune scores, and grouped according to BMI into two groups: an obese group (n = 38) and a normal group (n = 36). Kruskal-Wallis (KW) analysis was performed according to the scoring results of the grouped samples at *P* > 0.05 (**Supplementary Figure 1**). There were no significant differences in the results between the two groups, which is consistent with the fact that our two groups were from patients with cancer. KW analysis of other factors and obesity in the sample information from the GSE24185 data showed that menstrual status and HER2 status were associated with obesity (**Supplementary Table 1**). We selected 74 samples from the GSE24185 data set, all of which were female patients. The detailed information about age, classification, and menopausal status is shown in **Table 1** and **Supplementary Table 2**.

**Table 1:** Sample information in the GSE24185 dataset.

Characteristic	N=74 (%)
**Age**	
≥60	11 (14.8%)
<60	63 (85.1%)
**BMI status**	
normal	36 (48.6%)
obese	38 (51.3%)
**Grade**	
1/3	10 (13.5%)
2/3	23 (31.0%)
3/3	41 (55.4%)
**Menopause**	
PERI	8 (10.8%)
PRE	35 (47.2%)
POST	31 (41.8%)

### Screening of Differentially Expressed Genes (DEGs)

**Figure 1:**
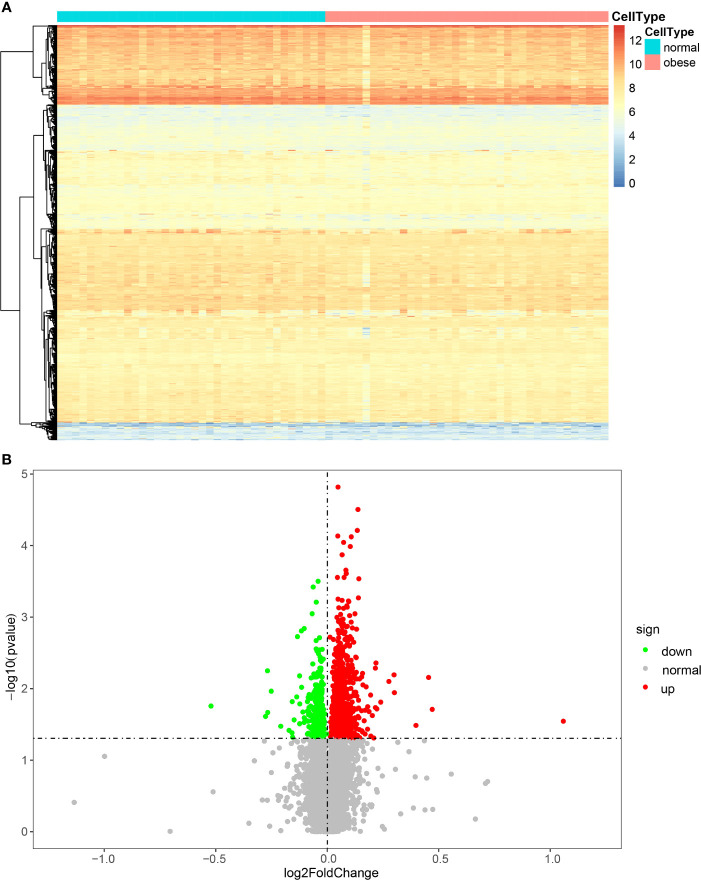
Heat map **(A)** and volcano map **(B)** of the expression of DEGs.

First, we conducted a PCA analysis and observed the degree of deviation between normal and obese samples (**Supplementary Figure 2**). We can see that there is no significant deviation between normal and obese samples, which is consistent with the fact that they are all cancer samples.The 74 samples from GSE24185 were divided into an obese and a normal group for the rank-sum test (Wilcox Test), and genes exhibiting significantly differential expression (P < 0.05) were screened. According to the differential gene expression analysis, 1173 genes were differentially expressed, of which 842 were upregulated and 331 were downregulated. To illustrate the distribution of each category, volcano maps and expression heat maps of differential genes were plotted (**Figures 1A, B**).

### Identification of Obesity-Associated Modules

**Figure 2:**
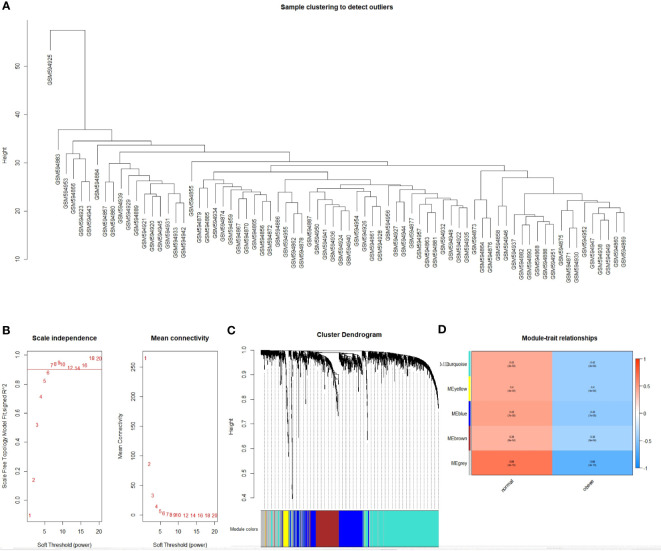
Identification of the functional modules associated with obesity. **(A)** Cluster dendrogram; **(B)** Analysis of network topologies for various soft threshold powers; **(C)** Clustering dendrogram of DEGs with dissimilarity based on topological overlap, together with assigned module colors; **(D)** Module-trait heatmap. Each row corresponds to a module eigengene, each column corresponds to a trait. Each cell contains the corresponding correlation and *P* value.

Coexpression analysis of the obtained DEGs were analyzed using the WGCNA software package. Data were first transformed into log2+1, and outliers were then processed. The GSM594925 sample was found to be in an outlier position, and it was removed from the analysis (**Figure 2A**). As shown in **Figure 2B**, the appropriate soft threshold values were screened out, and the resulting topological matrix was clustered using dissimilarity between genes. The tree was divided into modules (with a minimum of 30 genes per module) using the dynamic clipping method, and a total of five models were obtained (**Figure 2C**).

We plotted a heat map of module feature relationships to assess the association between each module and two clinical features (obese and nonobese). **Figure 2D** shows the correlation between the characteristic genes in a module and obesity traits. We performed co-expression analysis for DEGs, so grey module correlations were particularly high. This is because differentially screened genes are prone to form single or several genes with high correlation with traits, and cannot form effective gene modules. We selected the genes (blue module: r = 0.45, *P* = 8e-04 and turquoise module: r = 0.42, *P* = 2e-04) as the two gene modules exhibiting the best correlation, except for the grey module. The modules were then sorted to obtain 791 genes for further analysis.

### Functional Enrichment Analysis of Genes in Key Modules

In order to understand the functional differences of different modules, we conducted a difference analysis of the 5 module genes and performed GO analysis. The Blue module is mainly involved in “Golgi vesicle transport”, “protein secretion”, “response to oxidative stress” and other ways. The brown module is mainly related to “mesenchyme development”, “muscle tissue development”, “axonogenesis” and other pathways. The turquoise module is mainly related to “mRNA splicing”, “regulation of metabolic process”, “organic acid transport”, “regulation of neurotransmitter levels” and other pathways. And the turquoise module is also the group that has the most enriched GO pathways (**Supplementary Tables 3**–**5**); while the grey and yellow modules have not enriched any GO pathways.

**Figure 3:**
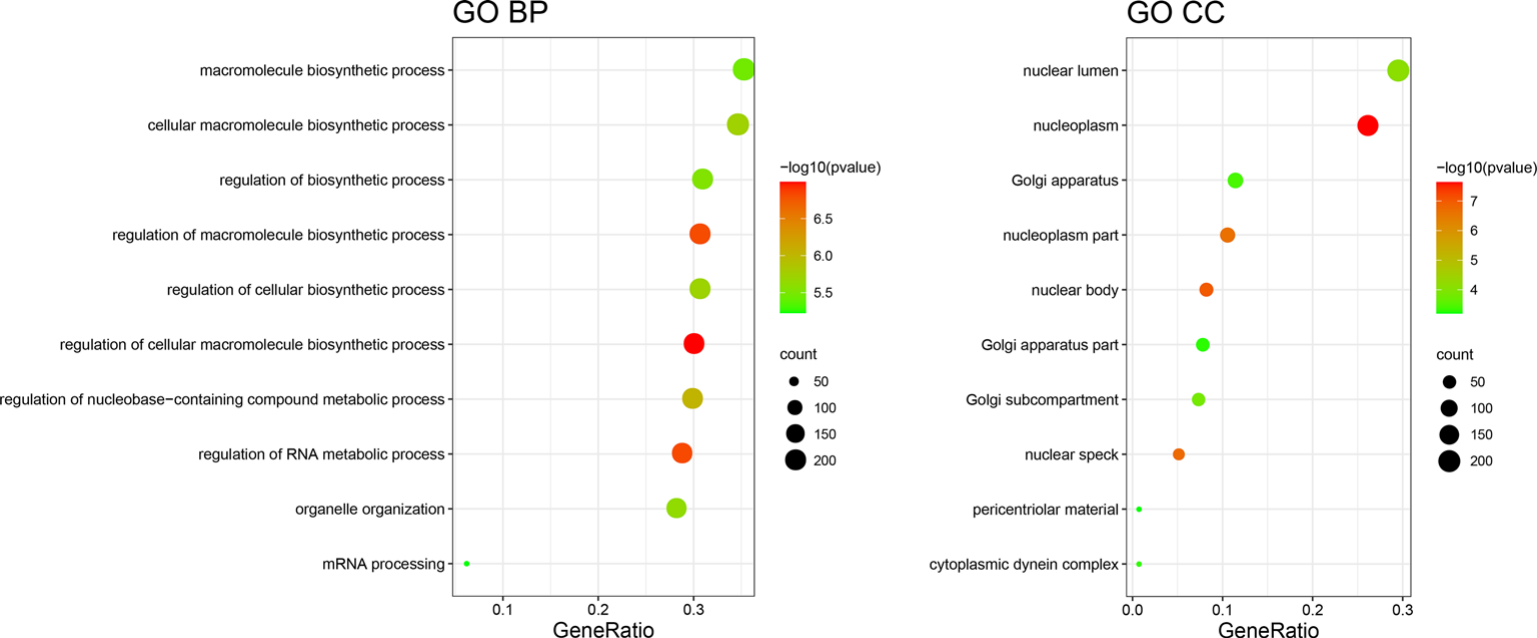
GO term enrichment results.

To further understand the biological function and pathway correlation of the blue and turquoise group modules, GO and KEGG pathway enrichment analyses were conducted. The results of the GO enrichment analysis showed that the modules were significantly enriched in the following terms: “regulation of cellular biosynthetic process,” “macromolecule biosynthetic process,” “DNA and nucleic acid binding.” Only two groups were enriched under MF entries, and KEGG results had only one pathway, so only the top 10 results of BP and CC were shown (**Figure 3**). **Figure 3** shows the top 10 enriched GO entries of the blue and turquoise group modules. **Supplementary Table 1** presents the results of all enriched genes according to the GO and KEGG enrichment analyses in detail.

### PPI Network Construction and Identification of Hub Genes

**Figure 4:**
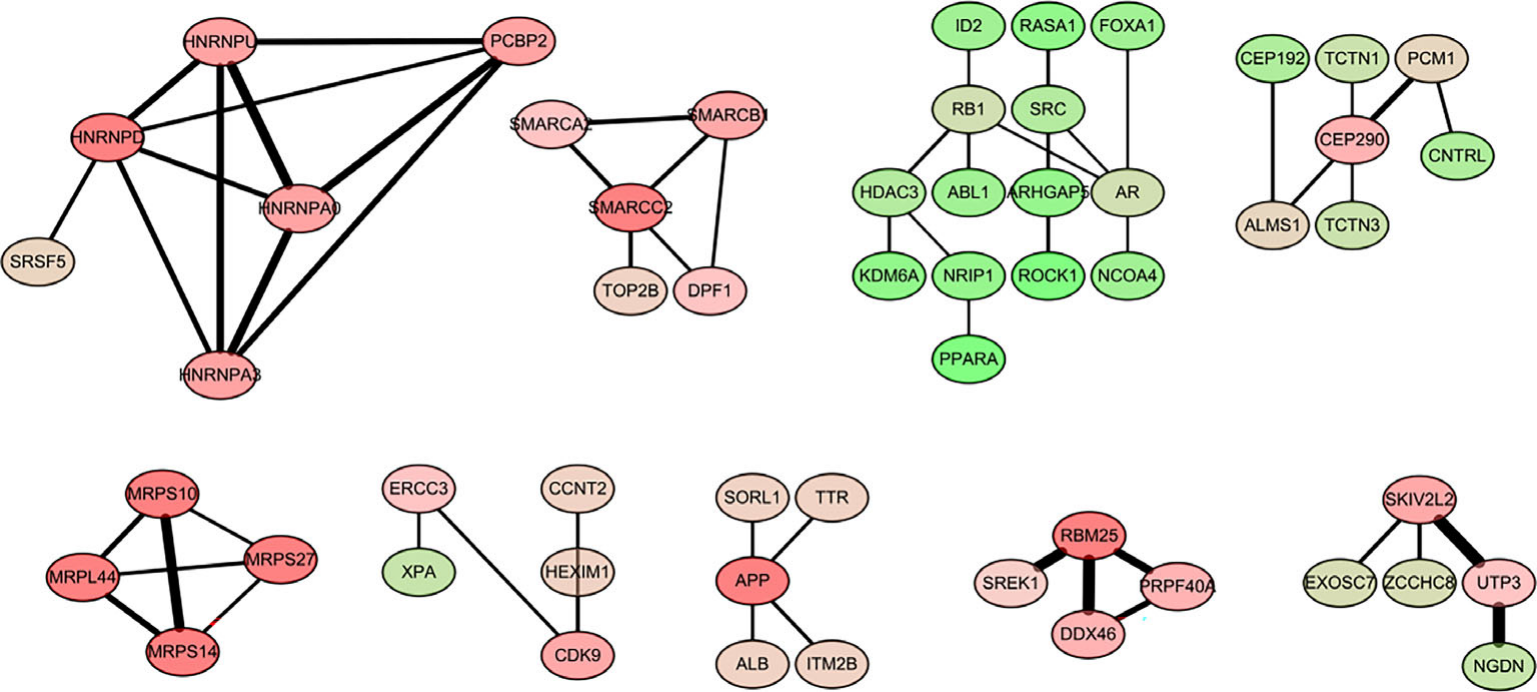
Visualization of the protein-protein interaction (PPI) network and the candidate hub genes.

The main objective of our study was to analyze the degree of correlation between genes in breast cancer and obesity and to determine the importance of genes in the different modules. The interaction network between proteins helps to mine the core regulatory genes. Using String online database (https://string-db.org/) and Cytoscape software were used to analyze the co-expressed genes. Among them were some interacting genes with high confidence, such as the potential interaction between mitochondrial ribosomal protein family genes (MRPS10, MRPS14, MRPS27, MRPL44), heterogeneous ribonucleoprotein family genes (HNRNPA0, HNRNPA3, HNRNPL),PCBP2). There was a potential interaction between PBM25, SREK1, PRPF40A, DDX46. A total of 55 genes, filtered into the PPI network (**Figure 4**).

### Prognostic Factors Were Screened by Univariate Cox Regression Analysis

We downloaded the expression profile data of these genes in breast cancer (including 1100 samples) from the TCGA database as well as the sample information table. Genes that had null expression values in all samples and 0 expression values in most samples were deleted. Finally, 740 genes were finally retained for univariate COX analysis. Univariate Cox analysis was performed for these 740 genes using the coxph function in the survival software package, and 30 potential prognosis-related genes were screened out (*p* value < 0.05; **Table 2**). Four of these genes were selected for survival analysis, and obvious survival differences can be seen between the two curves of each characteristic gene (**Supplementary Figure 3**).

**Table 2:** A total of 30 potential prognosis-related genes were screened by univariate analysis.

Gene	Beta	HR (95% CI for HR)	Wald Test	*p* value
ANOS1	0.36	1.4 (1-2)	4.8	0.029
BMERB1	-0.41	0.67 (0.47-0.94)	5.3	0.022
CACNA1D	-0.49	0.61 (0.43-0.87)	7.7	0.0056
CDC42EP3	-0.48	0.62 (0.43-0.89)	6.6	0.01
CFDP1	0.33	1.4 (1-1.9)	4	0.045
CHEK2	0.33	1.4 (1-1.9)	4.2	0.042
CIR1	0.45	1.6 (1.1- 2.2)	7.7	0.0055
CTBP2	-0.34	0.71 (0.51- 0.99)	4.2	0.042
DAO	0.37	1.4 (1- 2)	4.9	0.026
DBR1	0.34	1.4 (1-1.9)	4.3	0.038
DEF6	0.34	1.4 (1-1.9)	4.3	0.038
EEF1AKNMT	0.33	1.4 (1-1.9)	4	0.045
FASTKD2	0.39	1.5 (1.1- 2)	5.9	0.015
FCER1A	-0.41	0.66 (0.45- 0.97)	4.5	0.034
HRH3	0.72	2.1 (1.3- 3.3)	8.6	0.0033
KANSL2	0.33	1.4 (1- 1.9)	4.2	0.042
KHDC4	0.37	1.5 (1.1- 2)	5.1	0.023
LZTFL1	-0.35	0.7 (0.51- 0.97)	4.6	0.032
PARL	0.35	1.4 (1- 2)	4.6	0.033
PNOC	0.4	1.5 (1- 2.1)	4.9	0.026
PTPRCAP	0.35	1.4 (1- 2)	4	0.047
RBM4B	0.43	1.5 (1.1- 2.1)	7	0.0082
RHOG	0.39	1.5 (1.1- 2)	5.8	0.016
SELENBP1	0.35	1.4 (1- 2)	4.7	0.03
TCERG1	0.34	1.4 (1-1.9)	4.3	0.039
TCL1A	0.45	1.6 (1.1-2.3)	5.2	0.023
TP73-AS1	-0.34	0.71 (0.52 - 0.99)	4.2	0.041
TSPYL1	-0.45	0.64 (0.46 - 0.89)	7	0.0083
VNN1	0.46	1.6 (1-2.5)	4.4	0.037
ZCCHC8	0.41	1.5 (1.1-2.1)	6.5	0.011

### Lasso Regression Analysis

**Figure 5:**
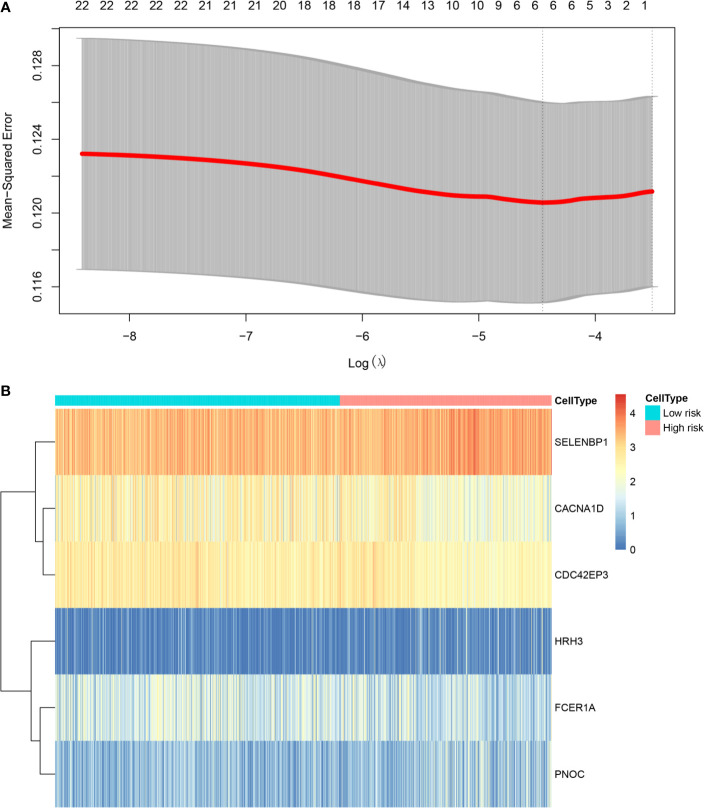
Prognostic risk assessment model. **(A)** Tenfold cross‐validation for tuning parameter selection in the LASSO model; **(B)** Heat map analysis of the gene expression of six pseudogenes in the high- and low-risk groups.

To facilitate subsequent validation, we obtained 24 genes from the intersection of the above 30 genes with another set of genes from the TCGA database. Expression profile data and survival analysis of these 24 genes were analyzed by lasso regression using glmnet package. We selected six pseudogenes with independent prognostic values: *SELENBP1*, *CACNA1D*, *CDC42EP3*, *HRH3*, *FCER1A*, and *PNOC*. We extracted the expression values of six characteristic genes and divided the samples into low- and high-risk groups (**Figure 5**).

### Difference of Immune Infiltration Between High- and Low-Risk Score Groups

**Figure 6:**
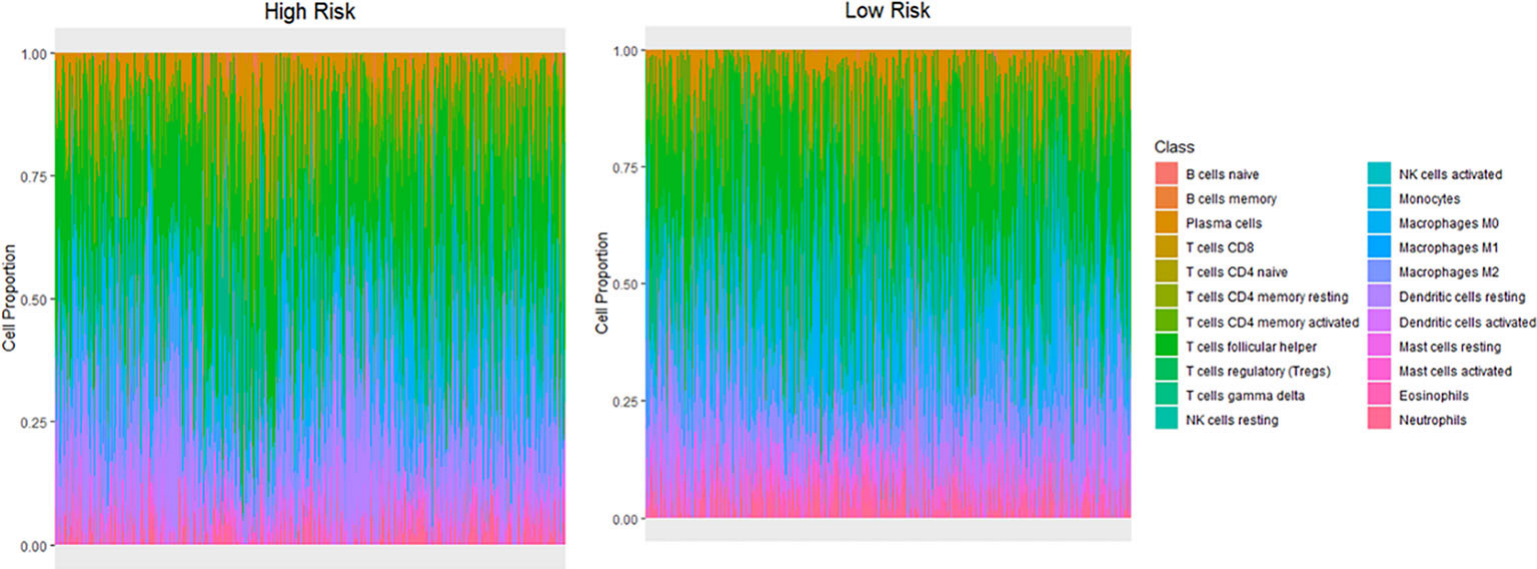
Differences in infiltration of different immune cell types between the high- and low-risk groups.

The Cibersort algorithm was used to calculate immune infiltration of 1100 samples for 22 types of immune cells. **Figure 6** presents a comparison of immune infiltration in the high- and low-risk groups (**Figure 6**). According to the results of rank-sum test (**Table 3**), revealed the presence of resting mast cells, eosinophils, CD8^+^ T cells, regulatory T cells (Tregs), naïve CD4 T cells, resting dendritic cells, neutrophils, naïve B cells, M0 macrophages, activated memory CD4^+^ T cells, activated dendritic cells, activated mast cells, resting memory CD4^+^ T cells, and memory B cells. There were differences in the proportion of infiltration of the 14 types of immune cells between the two groups.

**Table 3:** Rank-sum test of the proportion of each immune cell in the two groups.

Cell	*p* value
Resting mast cells	3.66E-18
Eosinophils	3.01E-12
CD8^+^ T cells	5.12E-08
Regulatory T cells (Tregs)	7.26E-08
Naïve CD4^+^ T cells	9.22E-06
Resting dendritic cells	1.19E-05
Neutrophils	2.85E-05
Naïve B cells	0.000205093
M0 macrophages	0.000572007
Memory activated CD4^+^ T cells	0.000847363
Activated dendritic cells	0.001242318
Activated mast cells	0.002280044
Resting memory CD4^+^ T cells	0.002988401
Memory B cells	0.032146684
M2 macrophages	0.063623725
Plasma cells	0.115015342
Monocytes	0.191466691
Gamma delta T cells	0.219624413
Resting NK cells	0.36141496
Follicular helper T cells	0.383084286
M1 Macrophages	0.621128772
Activated NK cells	0.826045073

### Relationship Between the Prognostic Model and Clinical Parameters

**Figure 7:**
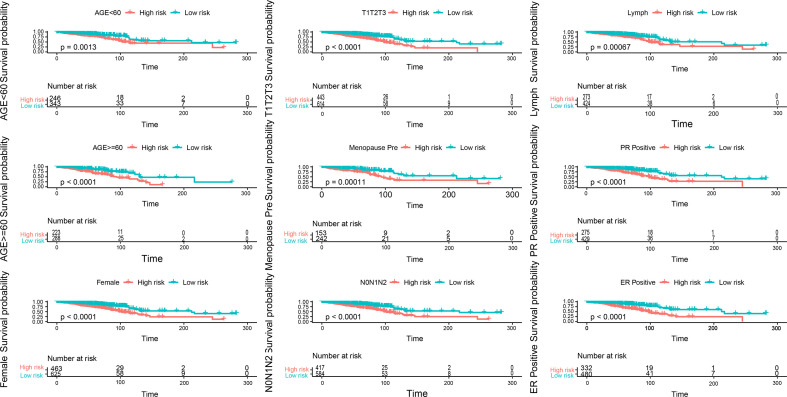
Evaluation of indicators related to breast cancer in the sample, blue is the low-risk group, light red is the high-risk group, each picture is a sample of different indicators.

The indicators related to breast cancer in the sample were evaluated according to the calculated risk value of each sample. As shown in the **Figure 7**, the prediction model constructed in the present study has good stability provided there is sufficient data. The survival time and survival rate were both higher in the low-risk group than in the high-risk group under different conditions such as age, T-stage, n-stage, gender, menstrual status, ER status, PR status, and lymph condition. To further understand the relationship between the prognostic model and other clinical data, we conducted univariate and multivariate COX regression analyses of TCGA data under different factors. The results revealed that the P values of the PR status and N-type in our prognostic risk prediction model were <0.05, and the p value and beta-value of the model we constructed were still within a reasonable range (**Tables 4**, **5**). These results confirm the independent prognostic value of the risk score.

**Table 4:** Univariate COX regression analysis.

	Beta	HR (95% CI for HR)	Wald Test	p value
Risk_class	0.75	2.1 (1 - 4.4)	4.1	0.044
HER2-negative *vs* HER2-positive	-0.28	0.76 (0.3 - 1.9)	0.35	0.55
ER-negative *vs* ER-positive	-0.55	0.57 (0.32 - 1)	3.3	0.068
PR-negative *vs* PR-positive	-0.68	0.51 (0.29 - 0.9)	5.5	0.019
Age (≥60 *vs* <60 years)	0.43	1.5 (0.87 - 2.7)	2.2	0.14
T (T1–T2 *vs* T3–T4)	0.6	1.8 (0.76 - 4.4)	1.8	0.18
N (N0–N1 *vs* N2–N3)	1.3	3.7 (1.6 - 8.8)	9	0.0027
Menopause (M0 *vs* M1)	0.11	1.1 (0.63 - 2)	0.14	0.71

**Table 5:** Multivariate COX regression analysis.

	Beta	HR (95% CI for HR)	p value
risk_class	0.867648	2.3813 (1.0792 - 5.2544)	0.0317
HER2 negative *vs* positive	-0.286233	0.7511 (0.2892 - 1.9507)	0.5567
ER negative *vs* positive	0.004105	1.0041 (0.4157 - 2.4256)	0.9927
PR negative *vs* positive	-0.996517	0.3692 (0.1550 - 0.8794)	0.0244
Age (≥60 *vs* <60 years)	0.698043	2.0098 (0.9642 - 4.1892)	0.0625
T (T1–T2 *vs* T3– T4)	0.056496	1.0581 (0.4221 - 2.6523)	0.9041
N (N0–N1 *vs* N2–N3)	1.169733	3.2211 (1.2833 - 8.0849)	0.0127
Menopause (M0 *vs* M1)	0.489737	1.6319 (0.7923-3.3611)	0.1840

## Discussion

Obesity has seen an unprecedented growth in recent decades, and its impact on health has become increasingly evident. Esposito et al. (19) showed that the presence of metabolic syndrome was associated with a 52% increased risk of breast cancer (*P* < 0.01). Some studies have shown that metabolic syndrome is associated with higher recurrence and mortality in breast cancer patients (20–22). Metabolic syndrome is considered to be a prognostic factor in patients with breast cancer. Here, to better predict the prognosis of breast cancer we constructed a risk prediction model of obesity in combination with breast cancer. We selected a set of breast cancer sample data sets in the GEO database that included obesity data (determined by the BMI). The data were scored by the ESTIMATE method for immunity and matrix, and the dataset was divided into two groups: obese group and nonobese group, for K-W analysis (*P* < 0.05), to determine suitability of the samples for use in this study. DEGs in the validated samples were screened using the Wilcoxon rank-sum test.

WGCNA is used to construct a gene coexpression network, where co-expression modules can be identified using the WGCNA package in R language (15). WGCNA has many outstanding advantages over other methods as it explores the association between co-expression modules and clinical features, and the results have higher reliability and biological significance compared to other methods. In this study, we used the WGCNA method to construct 5 co-expression modules from 1173 DEGs in 74 samples, and we calculated the correlation between co-expression modules and obesity. The blue group and turquoise group modules, which are highly correlated with clinical characteristics, are considered key modules in exploring the association with obesity. GO and KEGG enrichment analysis and PPI network analysis were carried out, and a total of 55 genes were filtered into the PPI network, including 55 and nodes. Then, univariate COX analysis and lasso regression analysis were performed to identify six pseudogenes with independent prognostic value.

*SELENBP1* has been shown to be expressed at low levels in cancers such as renal cell carcinoma, lung adenocarcinoma (23), and breast cancer (24) and is generally predictive of poor clinical outcomes. In our results, *SELENBP1* expression was lower in breast cancer samples in the nonobese group than in the obese group. *CACNA1D* is believed to regulate cell firing, and is highly associated with prostate cancer (25). Relevant bioinformatics analysis also confirmed that *CACNA1D* was highly expressed in prostate cancer, breast cancer, colorectal cancer, gastric cancer, lung cancer, uterine cancer, and other cancers (26). In our study, *CACNA1D* expression was lower in breast cancer samples of nonobese patients than obese patients. Results of the GO and KEGG analyses revealed that *CACNA1D* was enriched in the GO entries “neurotransmitter transport” and “cellular localization” and in the KEGG entry “Herpes simplex virus 1 infection”, and may be a novel oncogene in cancer development. *In vivo* “admix” experiments with breast cancer cells demonstrated that *Cdc42EP3* is required for efficient tumor growth. *Cdc42EP3*/*BORG2* has been reported to be needed for matrix remodeling, invasion, angiogenesis, and the tumor growth-promoting abilities of cancer-associated fibroblasts (27). Studies have shown that *HRH3* plays an important role in promoting tumor invasion and metastasis. Its expression is upregulated in lung cancer tissues, and it is associated with poor prognosis of lung cancer patients (28, 29). In our study, *HRH3* expression was higher in breast cancer samples of nonobese patients than obese patients. The *HRH3* gene was enriched in the entries of “neurotransmitter transport” and “cellular localization” in GO analysis. The relationship between *FCER1A* gene variants and allergic diseases has been demonstrated in human studies (30, 31). Some studies have hypothesized about the possible mechanisms underlying the association between the *FCER1A* gene and breast cancer, and suggested that immune-stimulating conditions such as infectious diseases and allergies may actually confer susceptibility to breast cancer (32). In our study, *FCER1A* expression was higher in breast cancer samples of nonobese patients than of obese patients. Ablation of PNOCARC neurons protects from obesity. PNOC expression was found to be significantly upregulated in gliomas (33). In our study, *PNOC* expression was higher in breast cancer samples of nonobese patients than of obese patients.

Early clinical studies have shown that immune infiltration has a great impact on the clinical course of concentrated types of cancer (34, 35). The results of immune infiltration in the high- and low-risk groups showed that the infiltration proportion of 14 of 22 types of immune cells significantly differed between the two groups. Resting mast cells accounted for the highest proportion of immune cells in both the groups, and the proportion of activated mast cells were found to significantly differ between the two risk groups. Mast cells can secrete several factors for the regulation of cancer cell growth (36). Eosinophils and T cells also account for a large proportion. Relevant studies have shown that an increase in the number of eosinophils coexists with obesity, and there is a positive correlation between blood eosinophil count and body mass index (BMI) or metabolic syndrome (37, 38). Therapeutic interventions targeting eosinophils in adipose tissue may have the potential to reduce inflammation and body fat while improving metabolic dysfunction in obese patients (39). Obesity-promoted breast tumor development is associated with loss of functional CD8^+^ T cells (40).

Patients in the high-risk group had a poor prognosis, and their survival time decreased with increase in the risk value. Under a range of different conditions, including age, T stage, N stage, sex, menstrual status, ER status, PR status, and lymph status, the survival time and survival rate were higher in the low-risk group than in the high-risk group. We also observed that PR status and N-typing remained important and independent risk factors for long-term survival. In both the univariate and multivariate COX regression analysis, the model constructed in the present study was found to be a good predictor of the prognosis, further indicating the prognostic value of this model.

In this study, the combination of WGCNA and the Cox proportional hazard model achieved reliable results in the identification of a co-expression network associated with survival and the construction of a risk score model. Our study provides potential models and biomarkers for further immune-related work and personalized drug treatment of breast cancer in breast cancer patients with obesity. However, this study also has some limitations. This is a retrospective study, and the predictive value of this model for prognosis has not been confirmed experimentally in clinical samples and the number of patients in the study type is limited. Finally, *in vivo* and *in vitro* experiments are needed to validate the findings of our study and to elucidate the molecular mechanisms underlying the roles of these genes in breast cancer patients with obesity.

## Data Availability Statement

Publicly available datasets were analyzed in this study. This data can be found here: GSE24185 dataset at https://www.ncbi.nlm.nih.gov/geo/query/acc.cgi?acc=GSE24185: TCGA dataset at https://xena.ucsc.edu/public/.

## Ethics Statement

Ethical review and approval was not required for the study on human participants in accordance with the local legislation and institutional requirements. Written informed consent for participation was not required for this study in accordance with the national legislation and the institutional requirements.Written informed consent was not obtained from the individual(s) for the publication of any potentially identifiable images or data included in this article.

## Author Contributions

NS, DM, YZ, and XQ conceived and designed the analysis and implemented the experimental studies. NS performed statistical analysis, interpreted results, graphed data, and wrote the paper. PG, YL, ZY, FH, and ZP modified the draft. YZ and XQ approved the draft of the paper. All authors contributed to the article and approved the submitted version.

## Funding

This work was supported by the Project of National Key Clinical Specialty Construction (413F1Z113), the Natural Science Foundation of Chongqing (cstc2018jcyjA0317), Military Medical Staff Innovation Plan of Army Medical University (No. XZ-2019-505-042) and Military Medical Staff Innovation Plan of Southwest Hospital (No. SWH2018BJLC-04).

## Conflict of Interest

The authors declare that the research was conducted in the absence of any commercial or financial relationships that could be construed as a potential conflict of interest.

## Publisher’s Note

All claims expressed in this article are solely those of the authors and do not necessarily represent those of their affiliated organizations, or those of the publisher, the editors and the reviewers. Any product that may be evaluated in this article, or claim that may be made by its manufacturer, is not guaranteed or endorsed by the publisher.

## Supplementary Material

Supplementary Figure 1 Stromal and immune scores.

Supplementary Figure 2 The principal component analysis(PCA) results for GSE24185 dataset.

Supplementary Figure 3 Prognostic value of differentially expressed genes in breast cancer patients.

Supplementary Table 2 Information of each sample in the GSE24185 dataset

Supplementary Table 3 The results of the GO term enrichment of the differential genes of the blue module.

Supplementary Table 4 The results of the GO term enrichment of the differential genes of the brown module.

Supplementary Table 5 The results of the GO term enrichment of the differential genes of the turquoise module.
