# Supplementary figures and images for: Construction of a Prognostic Risk Prediction Model for Obesity Combined With Breast Cancer
Source: Front Endocrinol (Lausanne). 2021 Sep 9;12:712513. doi: 10.3389/fendo.2021.712513 (PMC8458964; doi:10.3389/fendo.2021.712513)

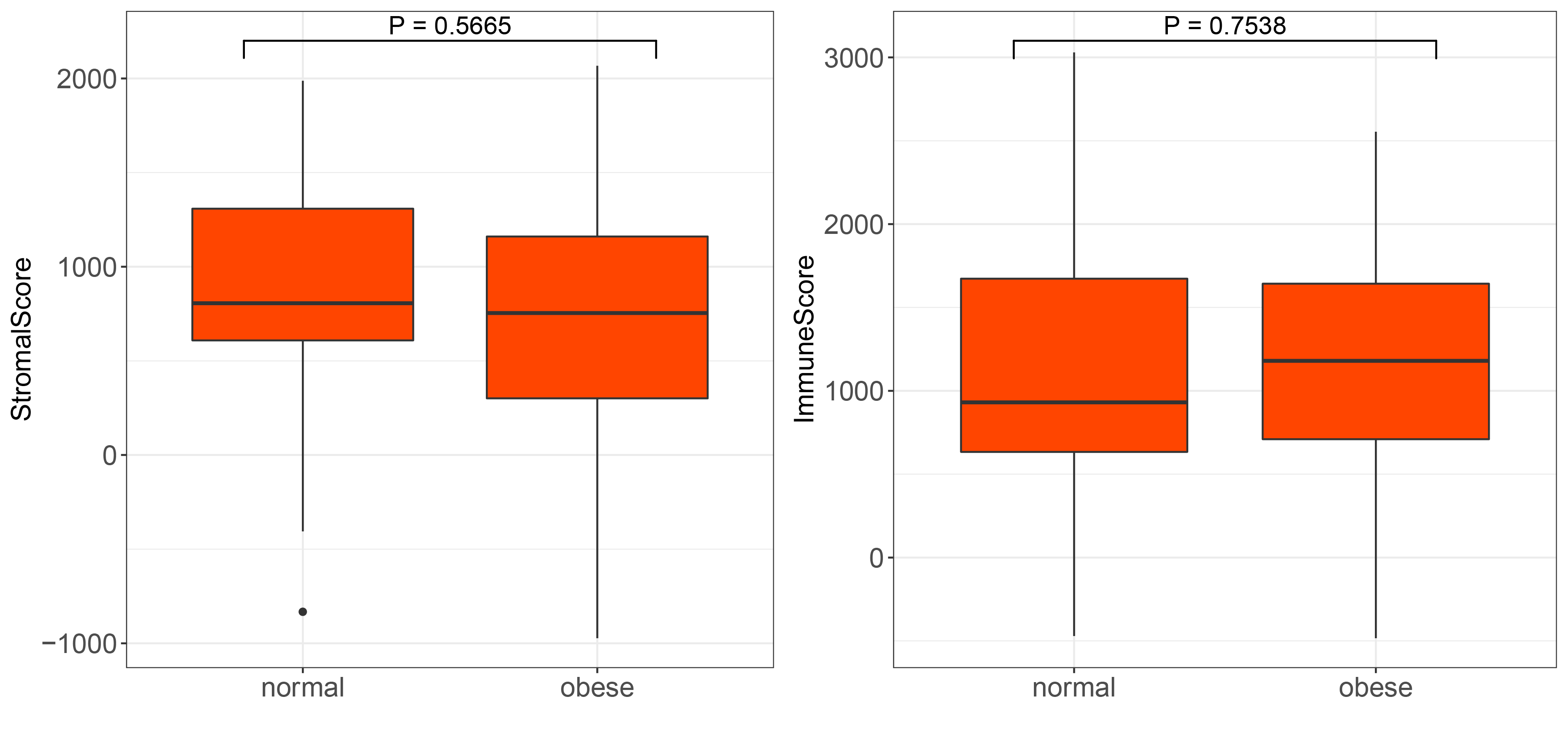

Supplement: Supplementary Figure 1 — Stromal and immune scores. [file Image_1.tif]

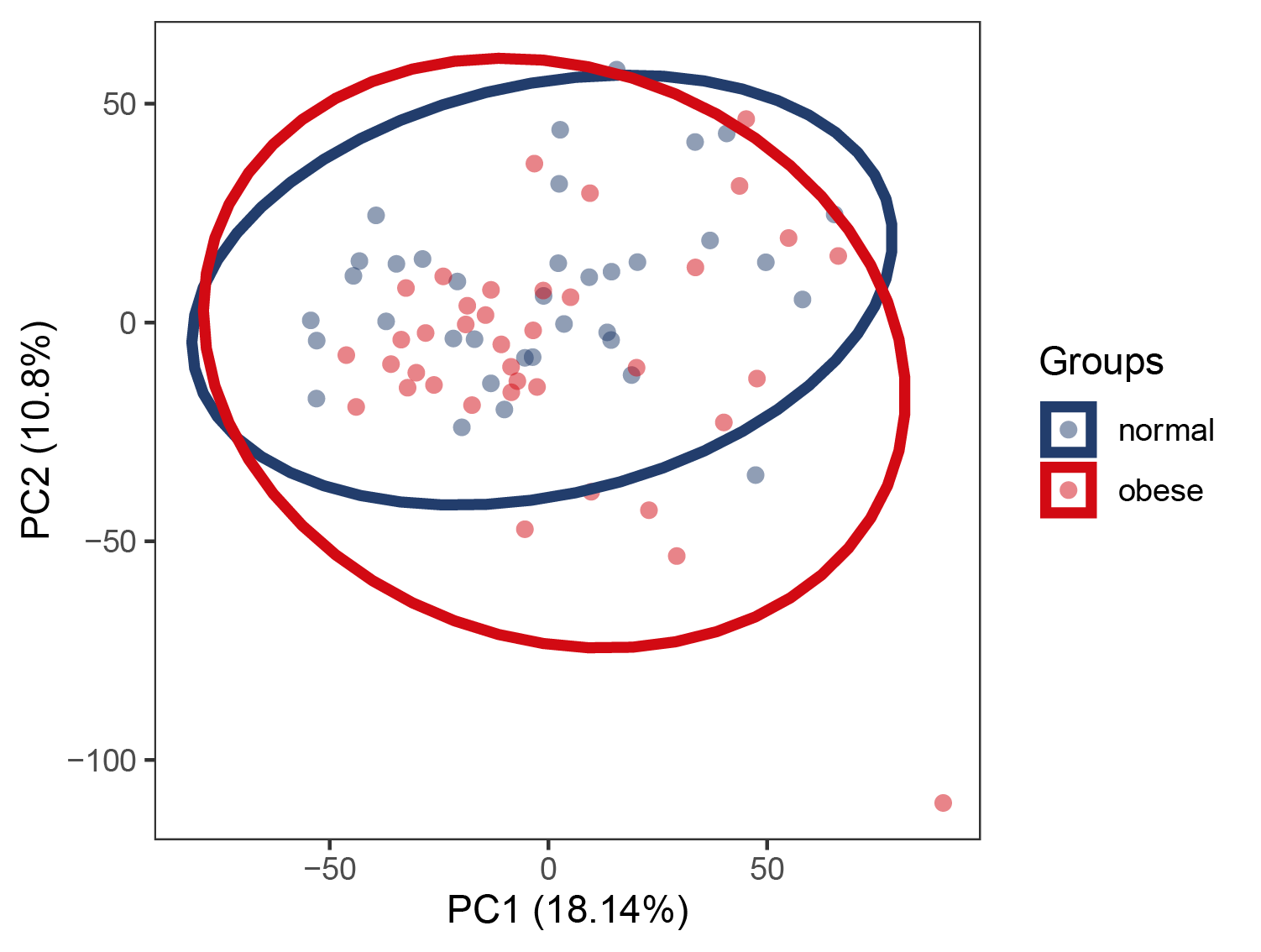

Supplement: Supplementary Figure 2 — The principal component analysis(PCA) results for GSE24185 dataset. [file Image_2.tif]

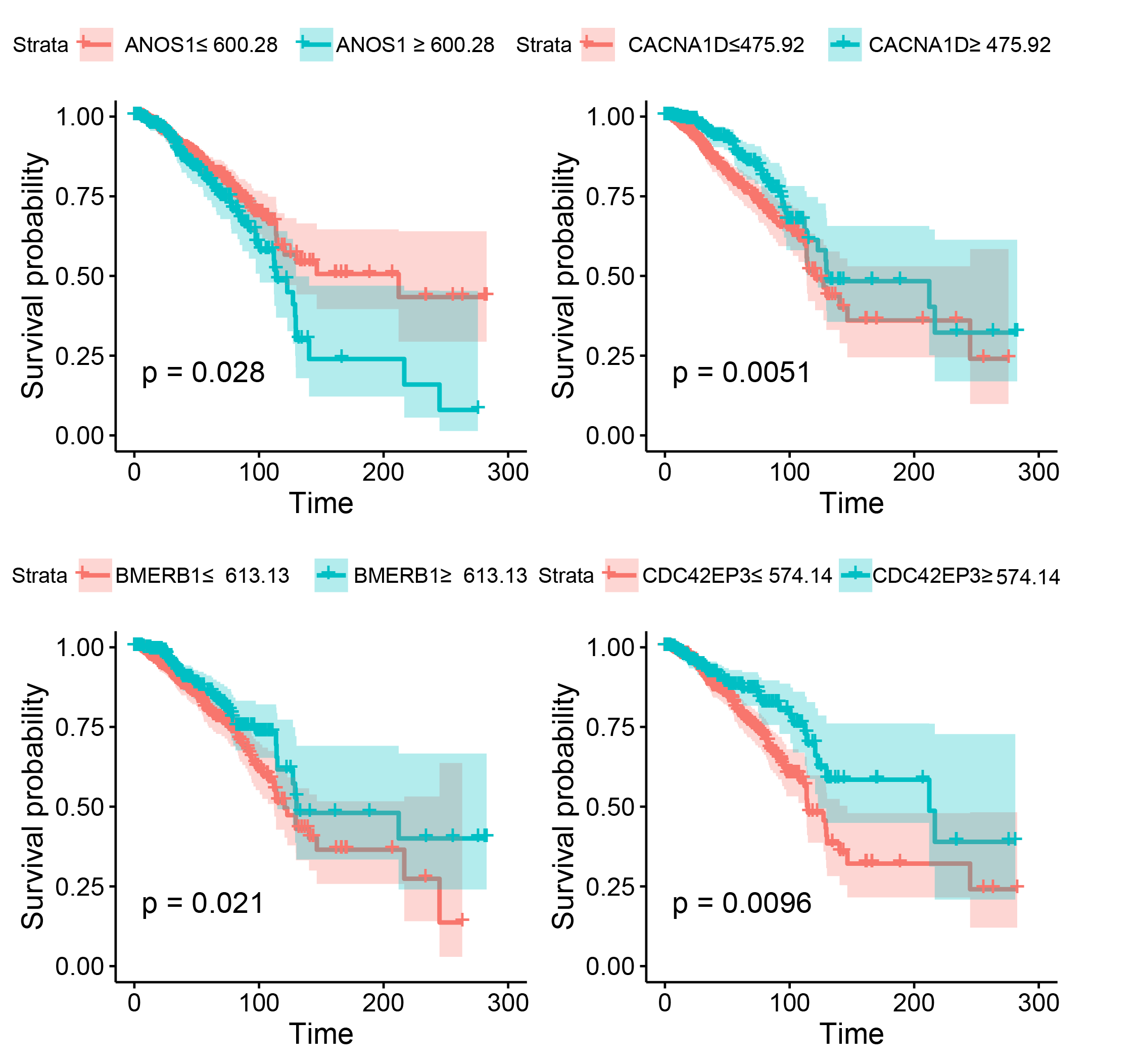

Supplement: Supplementary Figure 3 — Prognostic value of differentially expressed genes in breast cancer patients. [file Image_3.tif]
