# Supplementary material for: Construction of a Prognostic Risk Prediction Model for Obesity Combined With Breast Cancer
Source: Front Endocrinol (Lausanne). 2021 Sep 9;12:712513. doi: 10.3389/fendo.2021.712513 (PMC8458964; doi:10.3389/fendo.2021.712513)
Supplement: Supplementary file 8 [file Table_1.docx]

Supplementary Table.1 Association of other factors with obesity

| Variable | Nonobese | Obese | *P* value |
| --- | --- | --- | --- |
| No. of patients | 36 | 38 |  |
| **Age** |  |  | 0.192 |
| <40 | 8 | 4 |  |
| 40-50 | 13 | 15 |  |
| 50-60 | 10 | 13 |  |
| 60-70 | 4 | 6 |  |
| >70 | 1 | 0 |  |
| **Grade** |  |  | 0.1168 |
| I | 7 | 3 |  |
| II | 12 | 11 |  |
| III | 17 | 24 |  |
| **Lymph nodes** |  |  | 0.507 |
| + | 3 | 5 |  |
| - | 33 | 33 |  |
| **ER** |  |  | 0.6115 |
| - | 13 | 16 |  |
| + | 22 | 21 |  |
| Unknown | 1 | 1 |  |
| **PR** |  |  | 0.4219 |
| - | 15 | 19 |  |
| + | 19 | 18 |  |
| Unknown | 2 | 1 |  |
| **HER2** |  |  | 0.01494 |
| - | 15 | 25 |  |
| + | 4 | 6 |  |
| Unknown | 17 | 7 |  |
| **Menopause** |  |  | 0.0251 |
| Pre | 21 | 14 |  |
| Peri | 1 | 7 |  |
| Post | 14 | 17 |  |
